# Transforming growth factor β3 deficiency promotes defective lipid metabolism and fibrosis in murine kidney

**DOI:** 10.1242/dmm.048249

**Published:** 2021-10-01

**Authors:** Elia Escasany, Borja Lanzón, Almudena García-Carrasco, Adriana Izquierdo-Lahuerta, Lucía Torres, Patricia Corrales, Ana Elena Rodríguez Rodríguez, Sergio Luis-Lima, Concepción Martínez Álvarez, Francisco Javier Ruperez, Manuel Ros, Esteban Porrini, Mikael Rydén, Gema Medina-Gómez

**Affiliations:** 1 Lipobeta group, Área de Bioquímica y Biología Molecular, Departamento de Ciencias Básicas de la Salud, Facultad de Ciencias de la Salud, Universidad Rey Juan Carlos, Avda. de Atenas s/n, Alcorcón, 28922 Madrid, Spain; 2 Hospital Universitario de las Islas Canarias, Unidad de Investigación, 38320 La Laguna, Tenerife, Spain; 3 IIS-Fundación Jiménez Díaz, Departamento de Nefrología e Hipertensión, 28040 Madrid, Spain; 4 Departamento de Anatomía y Embriología, Facultad de Medicina, Universidad Complutense de Madrid, 28040 Madrid, Spain; 5 Centro de Metabolómica y Bioanálisis (CEMBIO), Facultad de Farmacia, Universidad San Pablo-CEU, CEU Universities, Urbanización Montepríncipe, Boadilla del Monte, 28660 Madrid, Spain; 6 Universidad La Laguna, Instituto Tecnologías Biomédicas (ITB), 38200 La Laguna, Tenerife, Spain; 7 Department of Medicine, Karolinska Institutet, Karolinska University Hospital, Huddinge, 141 86 Stockholm, Sweden; 8 LAFEMEX laboratory, Área de Bioquímica y Biología Molecular, Departamento de Ciencias Básicas de la Salud, Facultad de Ciencias de la Salud, Universidad Rey Juan Carlos, Avda. de Atenas s/n, Alcorcón, 28922 Madrid, Spain

**Keywords:** TGFβ, CKD, Fibrosis, Mitochondria, Lipid metabolism, Omics

## Abstract

Glomerulosclerosis and tubulointerstitial fibrosis are pathological features of chronic kidney disease. Transforming growth factor β (TGFβ) is a key player in the development of fibrosis. However, of the three known TGFβ isoforms, only TGFβ1 has an established role in fibrosis, and the pathophysiological relevance of TGFβ2 and TGFβ3 is unknown. Because *Tgfb3* deficiency in mice results in early postnatal lethality, we analyzed the kidney phenotype of heterozygous *Tgfb3-*knockout mice (*Tgfb3*^+/**−**^) and compared it with that of matched wild-type mice. Four-month-old *Tgfb3*^+/−^ mice exhibited incipient renal fibrosis with epithelial–mesenchymal transition, in addition to glomerular basement membrane thickening and podocyte foot process effacement associated with albuminuria. Also evident was insulin resistance and oxidative stress at the renal level, together with aberrant renal lipid metabolism and mitochondrial function. Omics analysis revealed toxic species, such as diacylglycerides and ceramides, and dysregulated mitochondrial metabolism in *Tgfb3*^+/−^ mice. Kidneys of *Tgfb3*^+/−^ mice showed morphological alterations of mitochondria and overactivation of non-canonical MAPK ERK1/2 and JNK cascades. Our study indicates that renal TGFβ3 might have antifibrotic and renoprotective properties, opposing or counteracting the activity of TGFβ1.

This article has an associated First Person interview with the first author of the paper.

## Abstract (precis)

**Summary:** Our study describes the renal abnormalities of heterozygous *Tgfb3-*targeted mice and suggests that TGFβ3 is renoprotective and may counteract the activity of TGFβ1.

## INTRODUCTION

Glomerulosclerosis and tubulointerstitial fibrosis are histopathological features of chronic kidney disease (CKD) from diverse etiologies. Fibrosis results from the imbalance between the synthesis and degradation of extracellular matrix (ECM), which leads to excessive ECM deposition and tissue scarring. The transforming growth factor β (TGFβ) family of cytokines are secreted multifunctional proteins involved in myriad biological processes such as cell proliferation and differentiation, wound healing and fibrosis. Three isoforms are known in mammals – TGFβ1, TGFβ2 and TGFβ3 – and are encoded by genes on different chromosomes. Because the three isoforms share 60–80% homology, their functional redundancy has long been assumed (Sureshbabu et al., 2016); however, studies in mice deficient in each of the specific isoforms have revealed non-overlapping phenotypes, indicating distinct biological functions (Kaartinen et al., 1995; Martínez-Sanz et al., 2008; Proetzel et al., 1995). The expression of the three isoforms has been confirmed in all renal cell types in human kidney: TGFβ2 and TGFβ3 are mainly expressed in podocytes, whereas TGFβ1 is predominantly expressed in mesangial cells and tubules (Sureshbabu et al., 2016). Of interest, both TGFβ1 and TGFβ3 signal through the type I (TIR) and type II (TIIR) TGFβ receptor, whereas TGFβ2 engages the type III TGFβ receptor (TIIIR) (Chaikuad and Bullock, 2016; Keiji and Kohei, 2008; Luo, 2017).

TGFβ1 is responsible for the activation of fibroblasts and their consequent differentiation to myofibroblasts, the major cellular players in fibrosis that produce and release collagens and other ECM components irrespective of the tissue or disease. The roles of TGFβ2 and TGFβ3 are less clear and remain controversial. For instance, TGFβ2 has been reported to have a predominantly profibrotic role in diabetic nephropathy (Lysaght, 2002; Wang et al., 2011; Yu et al., 2003), while other studies suggest an antifibrotic role in human podocytes (Papoutsoglou et al., 2019; Prelog et al., 2005; Ren et al., 2009; Sanford et al., 1997; Yu et al., 2003). Similarly, antifibrotic (Honardoust et al., 2012; Xu et al., 2014) and fibrotic (Murata et al., 1997) activities have been reported for TGFβ3. These contentious results might be explained by differences in the experimental approaches used, which include blocking the isoforms with antibodies, use of genetic knockouts or the addition of exogenous TGFβs, and by differences in which isoforms are being modified and to what extent. They may also depend on the cell type, as the application of TGFβ3, but not TGFβ1 or TGFβ2, to dermal wounds has antifibrotic effects (Shah et al., 1995), whereas the fibrotic effects of TGFβ3 and TGFβ1 were found to be additive in mesangial and tubular cells (Yu et al., 2003).

We recently identified TGFβ3 as a critical regulator of subcutaneous adipocyte proliferation upon changes in the expansion of white adipose tissue mass during obesity and weight loss (Petrus et al., 2018). The role of individual TGFβ isoforms in the kidney remains to be determined but might provide new insights into the development of therapeutic strategies to address CKD, ideally before its progression to an irreversible stage. *Tgfb3-*null mice are embryonic lethal (Kaartinen et al., 1995; Martínez-Sanz et al., 2008; Proetzel et al., 1995). In the present study, using a mouse heterozygous for a null mutation in *Tgfb3 (Tgfb3*^+/−^) (Petrus et al., 2018), we provide the first evidence that a decrease in *Tgfb3* expression in kidney, with no compensation by TGFβ1 or TGFβ2, is sufficient to produce a renal phenotype characterized by albuminuria, loss of function, fibrosis, epithelial–mesenchymal transition (EMT), and alterations in the lipid metabolism associated with mitochondrial dysfunction and oxidative stress. Our results lead us to propose that the balance between TGFβ isoforms in the kidney is important in the regulation/activation of downstream signaling pathways including ERK1/2 and JNK.

## RESULTS

### *Tgfb3*^+/−^ male mice present with albuminuria and impaired renal function

**Fig. 1.**
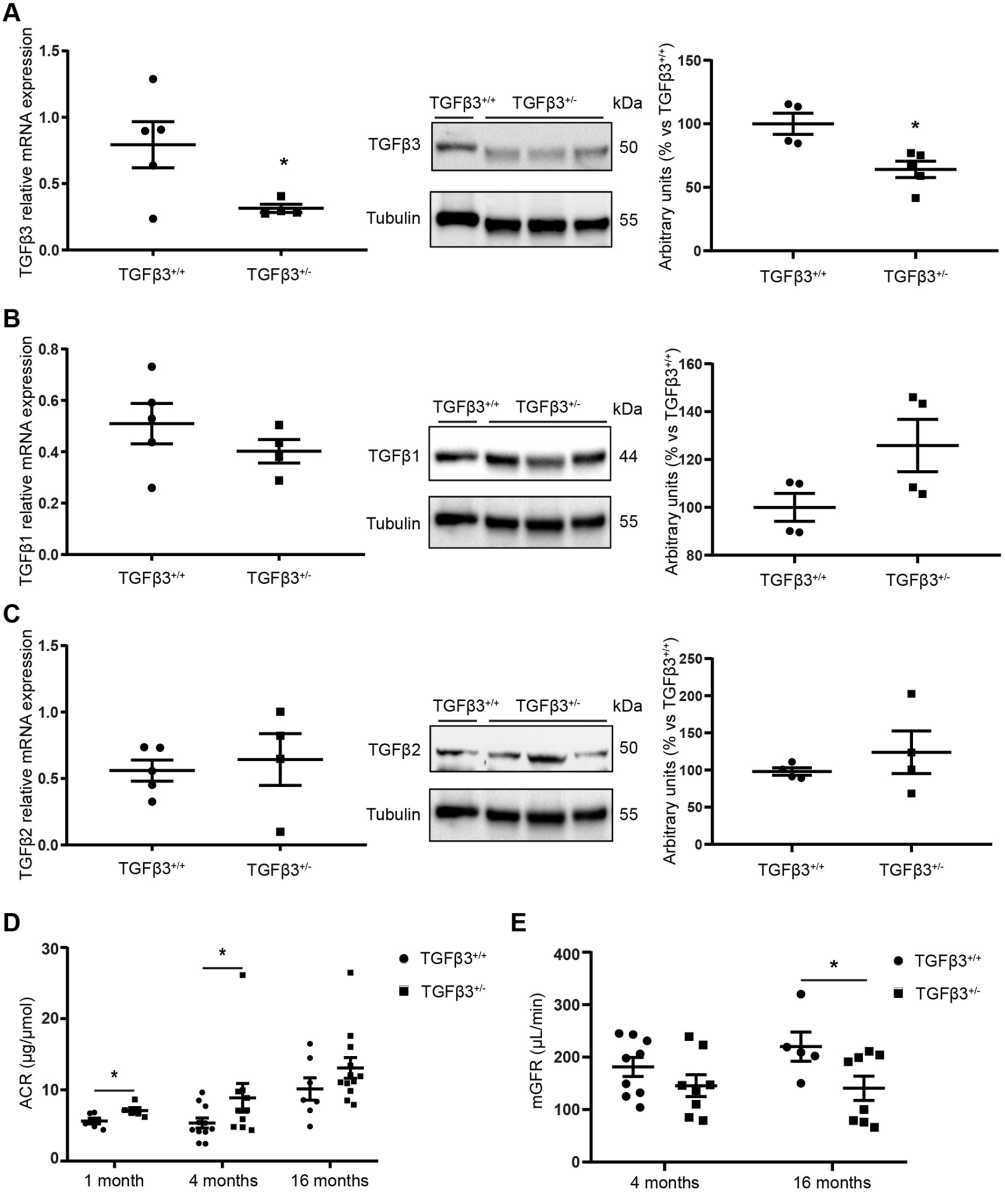
**Renal parameters in male *Tgfb3*^+/−^ and *Tgfb3*^+/+^ mice.** (A–C) Relative mRNA expression and western blot quantification in whole kidneys of male mice at 4 months of age of TGFβ3 (A), TGFβ1 (B) and TGFβ2 (C) (*n*=4–5; data are shown as mean±s.e.m.; unpaired Student's *t*-test with Welch's correction). (D) ACR at 1, 4 and 16 months of age (*n*=5–6 for the 1-month-old group, *n*=11–14 for the 4-month-old group and *n*=7–12 for the 16-month-old group; data are shown as mean±s.e.m.; unpaired Student's *t*-test was performed with Welch's correction for each age). (E) mGFR at 4 and 16 months of age (*n*=5–9; data are shown as median±interquartile range; Mann–Whitney U test was performed). *P<0.05 versus the control. ACR, albumin-creatinine ratio; mGFR, measured glomerular filtration rate.

Quantitative RT-PCR and western blot analysis of kidney extracts from male mice revealed that the expression of *Tgfb3* (mRNA and protein) in 4-month-old *Tgfb3*^+/−^ (heterozygous) mice was half that of their wild-type (*Tgfb3*^+/+^) counterparts (Fig. 1A), validating the genetic model. No compensatory changes were observed for the expression of *Tgfb1* and *Tgfb2* (Fig. 1B,C). We also measured the active and total TGFβ levels in both experimental groups and found that active TGFβ was ∼10% lower in *Tgfb3*^+/−^ mice than in *Tgfb3*^+/+^ mice, although not significantly (Fig. S1).

Analysis of the global phenotype of male *Tgfb3*^+/+^ and *Tgfb3*^+/−^ mice at 1, 4 and 16 months of age revealed no differences in total body weight or basal glucose levels between the *Tgfb3*^+/−^ and *Tgfb3*^+/+^ mice (Table 1; Table S4). As previously shown (Petrus et al., 2018), *Tgfb3*^+/−^ mice on chow diet had normal responses to glucose and insulin tolerance tests. Likewise, *Tgfb3*^+/−^ mice showed neither dyslipidemia nor hypercholesterolemia compared with *Tgfb3*^+/+^ mice (Table 1). The same was observed in females (Table S4).

**Table 1.**
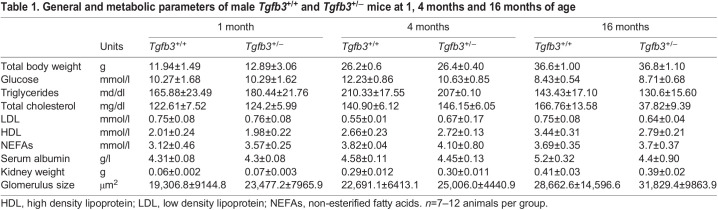
General and metabolic parameters of male *Tgfb3*^+/+^ and *Tgfb3*^+/−^ mice at 1, 4 months and 16 months of age

At the renal level, partial ablation of *Tgfb3* did not change kidney weight or glomerular area at 1, 4 or 16 months of age in male mice (Table 1). The same was observed in females at the same age (Table S4). Also, systolic blood pressure was not different between 4-month-old male *Tgfb3*^+/−^ and *Tgfb3*^+/+^ mice (117.26±3.8 versus 112.18±3.5 mm Hg).

To assess renal function, we evaluated urinary albumin as a critical feature of renal damage. We found a significant increase in the albumin/creatinine ratio (ACR) in male *Tgfb3*^+/−^ mice at 1 and 4 months of age, but not at 16 months of age (Fig. 1D). Although non-significant (*P*=0.11), we observed a 20% decrease in the measured glomerular filtration rate (mGFR) in 4-month-old male mice (Fig. 1E). *Tgfb3*^+/−^ male mice continued to lose renal function, and mGFR was significantly decreased by 40% at 16 months of age (Fig. 1E). Female *Tgfb3*^+/−^ mice did not show changes in albuminuria (Table S4).

### *Tgfb3* downregulation leads to renal fibrosis and changes in glomerular structure

**Fig. 2.**
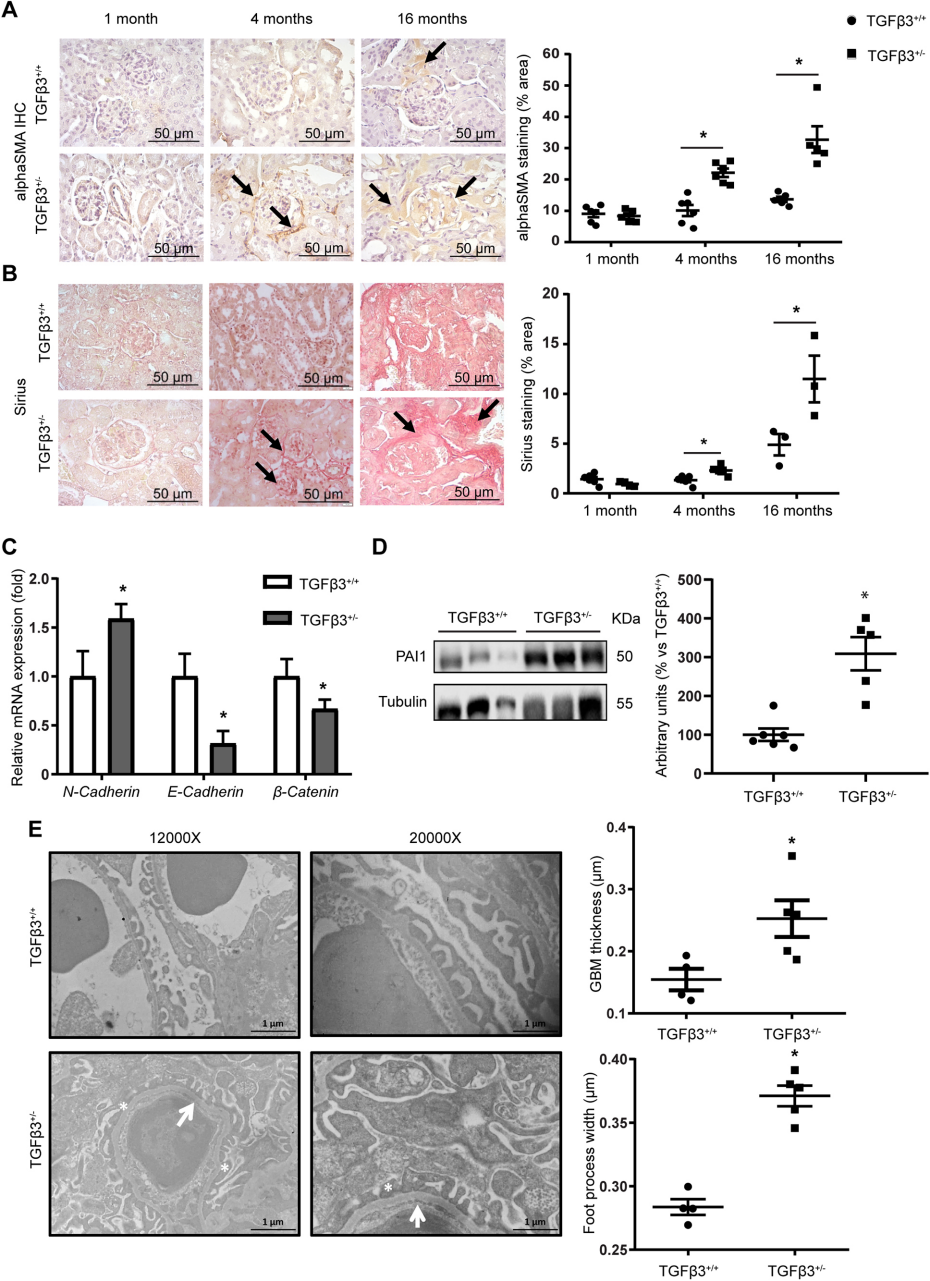
***Tgfb3*^+/−^ male mice show renal fibrosis at 4 months, but not 1 month, of age.** (A,B) α-SMA immunohistochemistry (*n*=5) (A) and Picrosirius Red staining (*n*=4–6) (B) in 1-, 4- and 16-month-old mice. Scale bars: 50 µm. (C) Relative mRNA expression of N-cadherin, E-cadherin and β-catenin in whole kidney of 4-month-old mice (*n*=5–6). (D) Representative western blot and densitometry of PAI-1 in whole kidney of 4-month-old mice (*n*=5–6). (E) GBM thickness and foot process width measured on transmission electron microscopy images in whole kidney of 4-month-old mice (*n*=4–5). Images were taken at a magnification of 400×. Black arrows mark positively stained areas of the tissue. White arrows mark thickened areas of the GBM, and white asterisks mark foot process effacement. Scale bars: 1 µm. Data are shown as mean±s.e.m.; unpaired Student's *t*-test with Welch's correction was performed. *P<0.05 versus the control. GBM, glomerular basement membrane; IHC, immunohistochemistry; PAI-1: plasminogen activator inhibitor-1; α-SMA, α-smooth muscle actin.

Histological analysis of kidney sections showed that the reduced levels of *Tgfb3* were associated with greater staining for α-smooth muscle actin (α-SMA), a marker of activated fibroblasts (myofibroblasts) at 4 months of age. These changes were not evident at 1 month of age (Fig. 2A). Myofibroblasts were preferentially located around the glomerular area and the interstice of proximal tubules. The area stained by α-SMA increased significantly in the kidneys of 16-month-old mice, both inside the glomeruli and in the proximal tubules (Fig. 2A). Furthermore, a marked interstitial and glomerular deposition of collagen fibers was observed in *Tgfb3*^+/−^ mice, as detected by Picrosirius Red staining (Fig. 2B). These changes were present in the kidneys of 4- and 16-month-old male mice but were not evident at 1 month of age. No fibrosis was observed in 4-month-old females (Fig. S2A,B); therefore, we decided from this point on to continue the study in 4-month-old males. Furthermore, we observed that the levels of the EMT genes *Cdh1* and *Ctnnb1* (E-cadherin and β-catenin) were lower, and the levels of *Cdh2* (N-cadherin) were higher, in whole kidneys of *Tgfb3*^+/−^ mice (Fig. 2C). This was accompanied by higher renal protein levels of plasminogen activator inhibitor-1 (PAI-1; also known as SERPINE1) (Fig. 2D), the primary inhibitor of metalloproteinases (MMPs), which are responsible for ECM degradation.

Electron microscopy analysis showed significant structural changes in the glomeruli of *Tgfb3*^+/−^ mice, including thickening of the glomerular basement membrane (GBM) and podocyte effacement of foot processes (Fig. 2E).

### *Tgfb3* downregulation results in lipid accumulation and lipid metabolism deregulation in the kidney

**Fig. 3.**
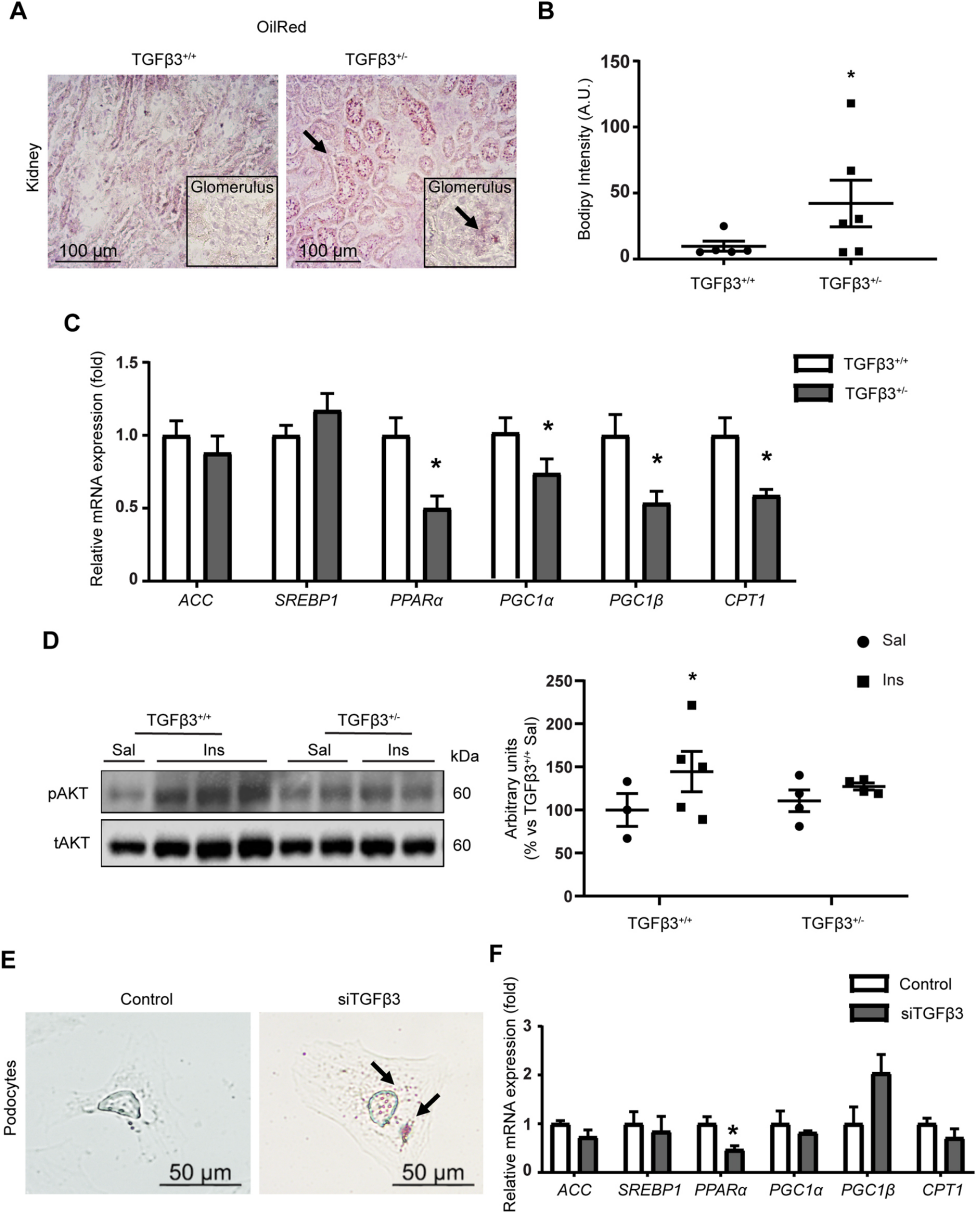
***Tgfb3* is involved in renal lipid metabolism.** (A) Oil Red O staining in kidney (200× magnification; glomerulus, 400× magnification). Scale bars: 100 µm. (B) Bodipy staining intensity measured by flow cytometry (*n*=5–6). (C) Relative mRNA expression of lipid metabolism genes in whole kidney (*n*=5–7). (D) Representative western blot and densitometry of phospho-AKT (*n*=4–5). (E) Oil Red O staining of siTGFβ3 podocytes. Images were taken at 400× magnification. Scale bars: 50 µm. (F) Relative mRNA expression of lipid metabolism genes in siTGFβ3 podocytes (*n*=3). All *in vivo* experiments were performed in 4-month-old male mice. Black arrows mark red dots representing lipid droplets. Data are shown as mean±s.e.m.; unpaired Student's *t*-test with Welch's correction was performed. *P<0.05 versus the control. ACC, acetyl-CoA carboxylase; AKT, protein kinase B; A.U., arbitrary units; CPT1, carnitine palmitoyltransferase 1; Ins, insulin; p, phospho; PGC1, peroxisome proliferator-activated receptor gamma coactivator 1; PPAR, peroxisome proliferator-activated receptor; Sal, saline; SREBP1, sterol regulatory element-binding protein 1; t, total.

At 4 months of age, whole kidneys of *Tgfb3*^+/−^ mice accumulated lipids, as assessed by Oil Red O and Bodipy staining (Fig. 3A,B). This accumulation was particularly evident in the tubules but was also observed inside the glomeruli (Fig. 3A). We then evaluated the expression of key genes involved in the synthesis and degradation of lipids. No significant differences were observed in the mRNA expression levels of genes involved in lipid synthesis, such as acetyl-CoA carboxylase (*Acc*; also known as *Acaca*) and sterol regulatory element-binding proteins (*Srebp1*; also known as *Srebf1*) in *Tgfb3*^+/−^ kidney samples; however, the mRNA levels of carnitine palmitoyltransferase 1 (*Cpt1*; also known as *Cpt1a*), involved in the transportation of lipid into the mitochondria, and other genes involved in fatty acid oxidation (FAO), such as the peroxisome proliferator-activated receptor α (*Ppara*), peroxisome proliferator-activated receptor gamma coactivator 1 α (*Pgc1a*; also known as *Ppargc1a*) and β (*Pgc1b*; also known as *Ppargc1b*) were decreased (Fig. 3C). We then tested for CD36 expression in the cell membrane to investigate whether the lipid accumulation is the cause or the consequence of the aberrant metabolism. No differences were observed in CD36 levels in whole kidney (Fig. S3A), suggesting that, when TGFβ3 levels are low, the observed lipid accumulation is not due to an increased import of fatty acids inside renal cells. The lipotoxicity caused by the accumulation of lipids in the kidney likely caused insulin resistance in *Tgfb3*^+/−^ mice, as suggested by the absence of AKT phosphorylation in kidney extracts (Fig. 3D). Based on this result, we tested for alterations in the levels of insulin receptor. A previous study in a mouse model of insulin receptor deletion in podocytes showed a similar phenotype to *Tgfb3*^+/−^ mice (Welsh et al., 2010). However, no differences in the levels of insulin receptor were observed at the podocyte level in our model (Fig. S3B).

To investigate further the link between TGFβ3 and lipid metabolism, we generated a knockdown cell line using siRNA against *Tgfb3* in podocytes (Fig. S4). We found that downregulation of *Tgfb3* led to a significant reduction in PPARα expression, which is involved in lipid metabolism, together with lipid accumulation, as evidenced by Oil Red O staining in podocytes (Fig. 3E,F). Furthermore, no differences in the levels of insulin receptor were observed in siRNA-silenced *Tgfb3* (siTGFβ3) compared with control podocytes (Fig. S5A). Similar results in lipid metabolism were observed in mouse proximal tubular renal epithelial cells (MCTs) (Fig. S5B,C).

**Fig. 4.**
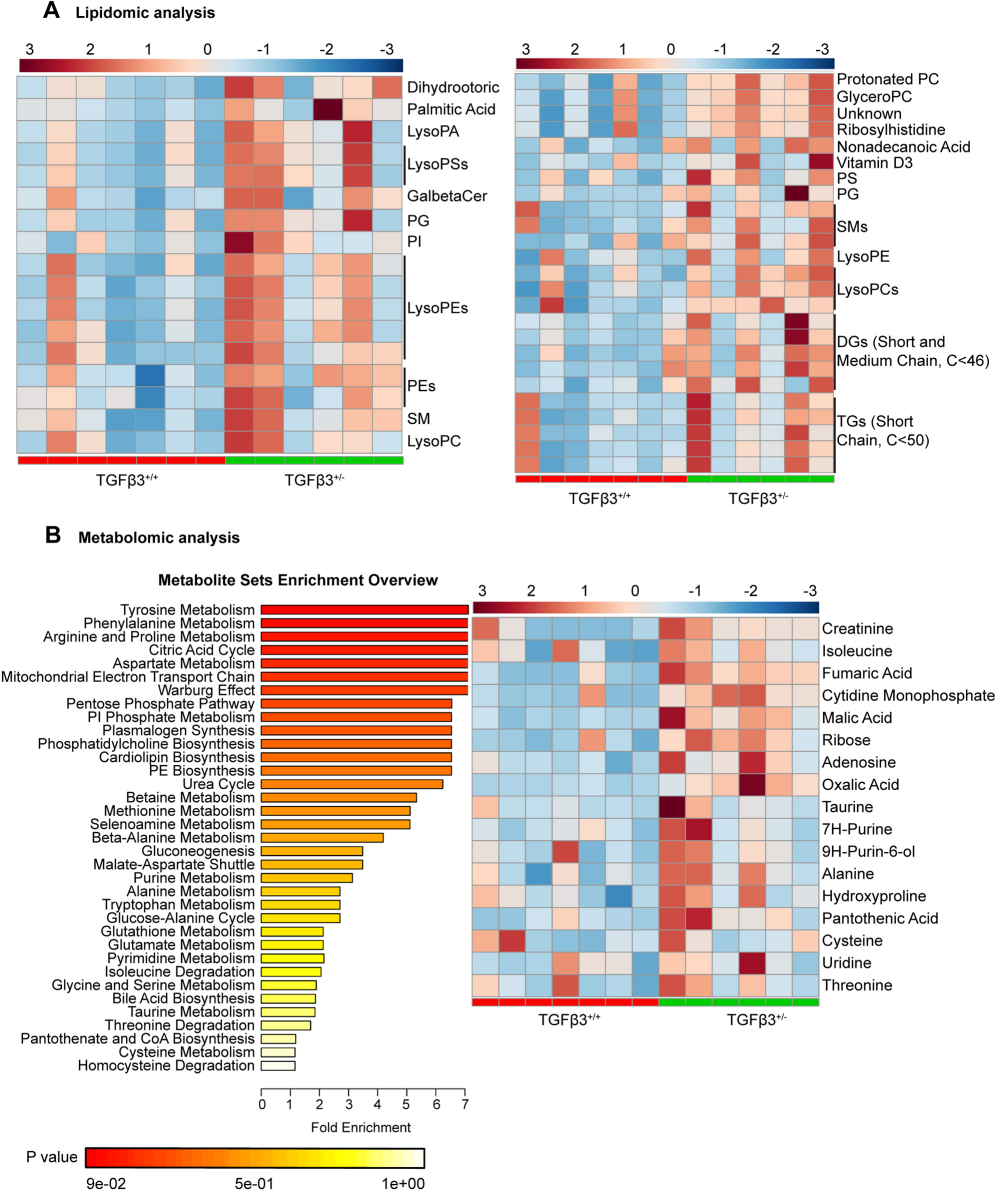
**Lipidomic and metabolomic analysis reveals a link between TGFβ3 and renal metabolism.** (A) Heatmap representations of lipidomic analysis performed in negative ionization mode {left, LC-MS [−]} and positive ionization mode {right, LC-MS [+]}. (B) Metabolomic analysis showing meaningful biological patterns identified in metabolite concentration through enrichment analysis (left) and heatmap representation (right). All experiments were done in whole kidney of 4-month-old male mice (*n*=6–7). Peak intensity for each individual was obtained applying univariate and multivariate tests (*P*≤0.05 or VIP>1). DG, diacylglycerol; GalBetaCer, galbetaceramide; GyceroPC, glycerophosphatidylcholine; LC-MS, liquid chromatography–mass spectrometry; LysoPA, lysophosphatidic acid; LysoPC, lysophosphatidylcholine; LysoPE, lysophosphatidylethanolamine; LysoPS, lysophosphatidylserine; PE, phosphatidylethanolamine; PG, phosphatidylglycerol; PI, phosphatidylinositol; PS, phosphatidylserine; siTGFβ3, siRNA-silenced *Tgfb3*; SM, sphingomyelin; TG, triglyceride; VIP, variable influence of projection.

We next investigated the chemical characteristics of the accumulated lipid species in *Tgfb3*^+/−^ kidney. Comparative lipidomic analysis revealed a significant increment in toxic lipid species, including palmitic acid, glycerophospholipids, sphingolipids including ceramide (42:1), diacylglycerol and medium-chain triglycerides in kidneys from *Tgfb3*^+/−^ compared with *Tgfb3*^+/+^ mice (Fig. 4A). We complemented this study with metabolomic analysis. Results showed a specific increase in fumaric and malic acid, adenosine and oxalic acid in *Tgfb3*^+/−^ compared with *Tgfb3*^+/+^ mice (Fig. 4B). Metabolite set enrichment analysis identified alterations in pathways of amino acids and lipid metabolism, including plasmalogens, cardiolipin and glycerophospholipids biosynthesis. Interestingly, from this enrichment overview, we found that the tricarboxylic acid cycle and the mitochondrial electron transport chain were among the most altered pathways (Fig. 4B).

### *Tgfb3*^+/−^ kidneys show mitochondrial alterations

**Fig. 5.**
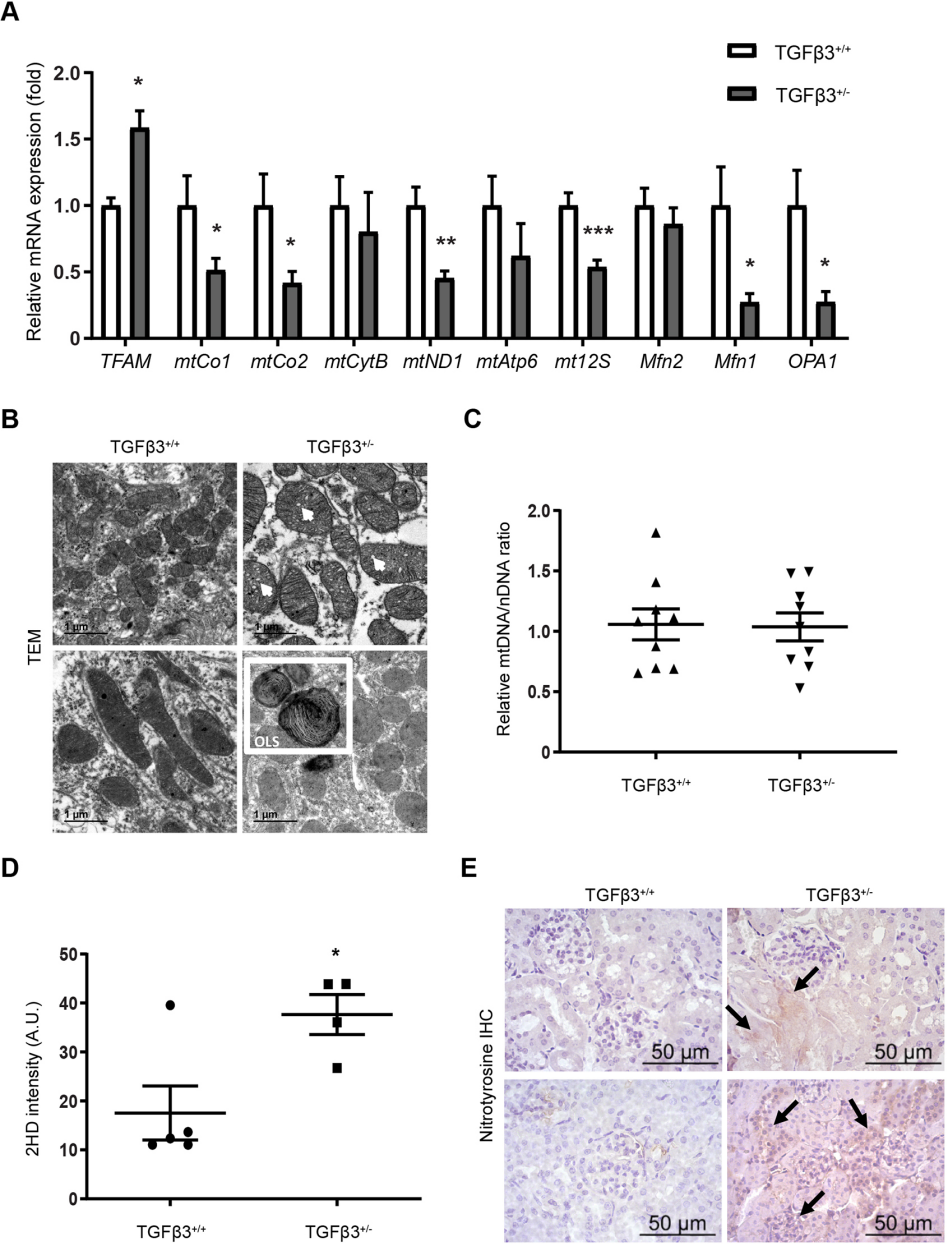
***Tgfb3* deficiency leads to mitochondrial dysregulation.** (A) Relative mRNA expression of mitochondrial and mitochondria-related genes (*n*=12). (B) Mitochondrial structural alterations by transmission electron microscopy. Scale bars: 1 µm. (C) Relative mtDNA/nDNA ratio (*n*=9). (D) Dihydroethidium staining intensity measured by flow cytometry (*n*=4–5). (E) Nitrotyrosine immunohistochemistry (*n*=6). Scale bars: 50 µm. All experiments were done in whole kidneys of 4-month-old male mice. Data are shown as mean±s.e.m.; unpaired Student's *t*-test with Welch's correction was performed. *P<0.05, **P<0.01 and ***P<0.001 versus the control. A.U., arbitrary units; IHC, immunohistochemistry; Mfn1, mitochondrial fusion protein type 1; Mfn2, mitochondrial fusion protein type 2; mtAtp6, ATP synthase membrane subunit 6; mtCo1, cytochrome oxidase subunit 1; mtCo2, cytochrome oxidase subunit 2; mtCytB, cytochrome B; mtDNA, mitochondrial DNA; mtND1, NADH dehydrogenase subunit 1; mt12S, mitochondrial 12S ribosomal gene; nDNA, nuclear DNA; OLS, onion-like shaped; OPA1, mitochondrial dynamin like GTPase; TEM, transmission electron microscopy; TFAM, mitochondrial transcription factor A; 2HD, dihydroethidium.

Our results thus far suggested a possible primary mitochondrial alteration in the kidneys of *Tgfb3*^+/−^ mice. Measurement of mitochondrial and mitochondrial-related genes in kidneys from 4-month-old male mice revealed a significant increase in the gene expression levels of mitochondrial transcription factor A (*Tfam*) in *Tgfb3*^+/−^ kidney, together with a decrease in cytochrome oxidase subunits 1 and 2 (*mtCo1* and *mtCo2*) and NADH dehydrogenase subunit 1 (*mtND1*), as well as a significant decrease in the mitochondrial 12S ribosomal gene (*mt12S*) (Fig. 5A). We also observed a significant decrease in the mRNA expression levels of mitochondrial fusion protein 1 (*Mfn1*) and the mitochondrial dynamin-like GTPase *Opa1* (Fig. 5A), which are involved in the structural maintenance of mitochondria. By contrast, no changes were observed in mitochondrial fusion protein type 2 (*Mfn2*) (Fig. 5A). Structural analysis by transmission electron microscopy showed the presence of onion-like-shaped mitochondria in kidneys from *Tgfb3^+/−^* mice, with some showing altered cristae structures (Fig. 5B). No differences were observed in the number of mitochondria calculated as mitochondrial DNA (mtDNA)/nuclear DNA (nDNA) ratio (Fig. 5C).

Given the mitochondrial phenotype, we studied the effect that the downregulation of *Tgfb3* could have on oxidative stress. As expected by the mitochondrial dysfunction observed, kidneys of *Tgfb3^+/−^* mice showed a significantly higher generation of reactive oxygen species (ROS) than did kidneys of *Tgfb3*^+/+^ mice (Fig. 5D). Moreover, levels of nitrotyrosine, a marker of nitrative and oxidative stress, were increased (Fig. 5E) in *Tgfb3^+/−^* compared with *Tgfb3*^+/+^ kidneys, particularly inside the glomeruli and around the proximal tubules.

### *Tgfb3* deficiency leads to overactivation of TIR–TIIR downstream pathways

**Fig. 6.**
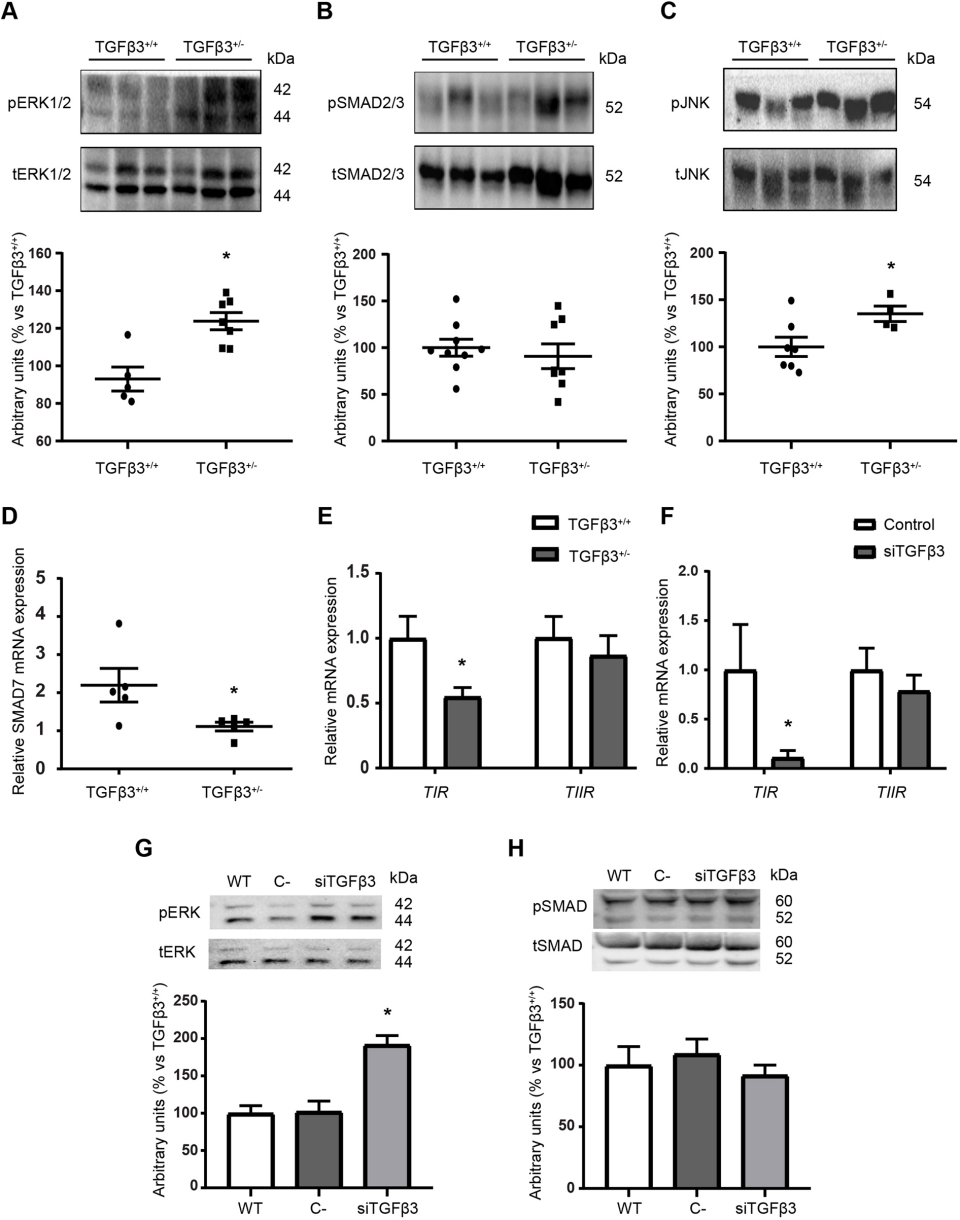
**Depletion of *Tgfb3* leads to higher phosphorylation of TIR–TIIR-mediated pathways.** (A–C) Representative western blots and densitometry of the ERK1/2 pathway (*n*=8–9) (A), SMAD2/3 pathway (*n*=8-9) (B) and JNK pathway (*n*=4-7) (C) in whole kidney. (D,E) Relative mRNA expression of SMAD7 (*n*=5) (D) and TIR and TIIR (*n*=4–6) (E) in whole kidney. (F) Relative mRNA expression of TIR and TIIR in siTGFβ3 podocytes. (G,H) Representative western blots and densitometry of the ERK1/2 pathway (G) and SMAD2/3 pathway (H) in siTGFβ3 podocytes. All *in vivo* experiments were done in whole kidney of 4-month-old male mice. Data are shown as mean±s.e.m.; unpaired Student's *t*-test with Welch's correction was performed. *P<0.05 versus the control. C−, negative control; ERK, extracellular signal-regulated kinase; JNK, c-Jun N-terminal kinase; p, phospho; siTGFβ3, siRNA-silenced *Tgfb3*; SMAD, mothers against decapentaplegic homolog; t, total; TIR, TGFβ type I receptor; TIIR, TGFβ type II receptor; WT, wild type.

As TGFβ3 levels are lower in *Tgfb3^+/−^* than in *Tgfb3*^+/+^ mice, we evaluated whether the downstream pathways of its receptors, which are shared with TGFβ1, were affected. Surprisingly, lower levels of TGFβ3 did not translate into lower activation of the downstream pathways. On the contrary, the non-canonical MAPK pathways ERK1/2 and JNK were significantly more phosphorylated in *Tgfb3^+/^*^−^ kidney than in *Tgfb3^+^*^/+^ kidney (Fig. 6A,C). No changes in the phosphorylation of the canonical SMAD2/3 pathway were observed (Fig. 6B). However, we observed lower *Smad7* mRNA levels, responsible for shutting down the SMAD2/3 cascade (Fig. 6D). We then examined for changes in the shared receptors, TIR and TIIR. Significantly lower mRNA levels of TIR were detected in *Tgfb3^+/^*^−^ kidney, whereas no differences were observed for TIIR (Fig. 6E). Interestingly, the same phenotype was observed in cultured podocytes upon *Tgfb3* downregulation using siRNA, suggesting that the lack of TGFβ3 could lead to the reduction in the levels of TIR and ERK1/2 overactivation (Fig. 6F–H).

## DISCUSSION

TGFβ3 is a TGFβ family protein that is assumed to behave in a similar manner to TGFβ1. In the present study, we established a potential renoprotective function of TGFβ3 using an *in vivo* genetically modified mouse model lacking one of the *Tgfb3* alleles (*Tgfb3*^+/−^), and corroborated some results in *Tgfb3-*knockdown cell lines. Male *Tgfb3*^+/−^ mice at 4 months of age exhibit renal fibrosis, EMT and glomerular damage with foot process effacement in podocytes, and show albuminuria, loss of renal function, lipid accumulation, lipid metabolism deregulation, insulin resistance and mitochondrial dysfunction with oxidative stress. Albuminuria and fibrosis are not observed in *Tgfb3*^+/−^ female mice at the same age. These results fit well with what is observed in humans: *TGFB3* polymorphisms lead to albuminuria in non-diabetic hypertensive men but not in women (Hu et al., 2008). The authors of the aforementioned study suggest that the polymorphism may be involved in the transcriptional activity of TGFβ3. Estrogens or their associated metabolites can ameliorate the renal damage associated with lack of TGFβ3 function, as occurs in other renal diseases (Gross et al., 2004). Indeed, it has been reported that TGFβ-stimulated collagen synthesis can be reversed with estradiol (Lei et al., 1998; Silbiger et al., 1998), and it has been suggested that the beneficial effects of estrogens on renal damage are, to some extent, attributable to the inhibition of TGFβ expression (Avouac et al., 2020). Moreover, estrogen treatment increases MMP activity and reduces renin angiotensin aldosterone system (RAAS) activation, likely ameliorating the fibrosis and the glomerular damage caused by hyperfiltration (Elliot et al., 2003). Our *Tgfb3*^+/−^ model is metabolically normal (non-diabetic, non-obese and non-hypertensive) (Petrus et al., 2018). These results suggest that the evident damage in the kidneys of *Tgfb3*^+/−^ mice is caused by the reduction in TGFβ3 levels and is independent of body weight, diabetes, dyslipidemia or hypertension. Because the strongest renal phenotype was observed in 4-month-old male mice, we focused most of this study on this group.

*Tgfb3*^+/−^ mice showed a 50% reduction in mRNA and protein levels of TGFβ3 in the whole kidney, and we would also expect to observe functional alterations at a global level. However, TGFβ3 downregulation may not affect all renal cell types in the same manner. Importantly, we did not observe any compensation in the levels of TGFβ1 and TGFβ2 or in the TGFβ active fraction.

TGFβ3 deficiency in male mice leads to incipient fibrosis in kidneys at 4 months of age but not at 1 month of age, suggesting that the disease evolves and worsens over time. Indeed, 16-month-old *Tgfb3*^+/−^ male mice showed significantly lower mGFR values than 4-month-old mice, which were accompanied by aberrant tubulointerstitial fibrosis, indicating worse renal outcomes despite no significant increase in the ACR. TGFβ3 seems to regulate ECM homeostasis both at the synthesis and degradation level. In the model used here, TGFβ3 deficiency promotes the overactivation of fibroblasts to myofibroblasts and an increase in EMT, leading to a higher production of ECM components and a strong inhibition of MMPs via increased PAI-1 levels. Myofibroblasts and collagen fiber accumulation were found to some extent inside the glomeruli, but mainly in the interstitial area of the proximal tubules and around the Bowman's capsule. It is worth noting that fibrosis was not evident in the tubules that are distal to glomeruli. Glomeruli and the areas close to the glomeruli are likely to be most affected by the lack of TGFβ3 because, as described (Ito et al., 2010), podocytes are the main renal cells that secrete TGFβ3. Therefore, the glomeruli and the surrounding areas would be the under the ‘area of influence’ of podocyte secretion. In fact, our analysis revealed significant alterations in glomerular structure linked to fibrotic processes such as GBM thickening and foot process effacement. These alterations in the glomerular structure are found in different renal diseases (D'Agati et al., 2016; Meng et al., 2015; Miner, 2011, 2012) and might explain, partly, the albuminuria and the 20% loss of renal function at 4 months of age, with a further decline to 60% of its normal mGFR (40% loss) in older animals. It is known that some epigenetic modifications are associated with the development of renal fibrosis (Lin and Wu, 2020; Rousselle et al., 2021) and that TGFβ stimulates epigenetic modifications (Dees et al., 2020). There is evidence in the literature linking TGFβ and renal disease through epigenetic modifications, especially in the context of miRNAs (Gomez et al., 2016; Kota and Kota, 2017). miRNA-mediated control of kidney fibrosis mainly occurs through the regulation of TGFβ1 signaling in a cell- and context-dependent manner, using the SMAD pathway as one of the main targets (Meng et al., 2015). For instance, miR-21 seems to be specifically involved in renal fibrosis regulation (McClelland et al., 2015). Also, in terms of lipid metabolism, it has been reported that miR-150 and miR-495 can reduce FAO-associated oxygen consumption and promote TGFβ1-induced fibrogenic transformation in the human renal tubular epithelial cell line HKC-8 (Miguel et al., 2021). However, as far as we know, no epigenetic mechanisms have been reported to specifically regulate *TGFB3* expression. This is an unexplored area that could shed light onto the link between TGFβ3 and renal fibrosis.

We recently showed that TGFβ3 stimulates adipocyte precursor proliferation and regulates fat cell number in subcutaneous adipose tissue (Petrus et al., 2018). Our present results show that TGFβ3 also plays a role in renal lipid metabolism. We observed the accumulation of neutral lipids by Oil Red O and Bodipy staining and decreased FAO, which is common in many renal diseases. Lipid accumulation seems to be concentrated in the proximal tubules, although glomerular deposits were also observed, which agrees with previous published studies (Bobulescu, 2010; Corrales et al., 2018). Studies in proximal tubular cells and podocytes with reduced levels of TGFβ3 (siTGFβ3) confirmed these results. In the two TGFβ3-knockdown cell lines, one of the genes significantly reduced upon TGFβ3 downregulation was *Ppara*, a master regulator of lipid metabolism. By contrast, we did not observe differences in the expression of the lipid transporter CD36 in renal cell membranes in whole kidney, suggesting that is the deregulation of lipid metabolism (likely produced by early downregulation of PPARα) that leads to lipid accumulation and the subsequent mitochondrial phenotype observed in the mouse model. Furthermore, the link between PPARα downregulation and lipid accumulation has previously been described and was shown to be directly related to the development of renal fibrosis (Chung et al., 2018). The toxic effects of lipid accumulation in the kidney, known as renal lipotoxicity, could be the cause of the insulin resistance that we observed in *Tgfb3*^+/−^ kidneys (Martínez-García et al., 2015; Opazo-Ríos et al., 2020). A previous study has indicated that insulin receptor deletion in podocytes can lead to a similar phenotype mirroring diabetic nephropathy in a normoglycemic environment (Welsh et al., 2010); however, we observed no differences in insulin receptor expression between *Tgfb3*^+/−^ and *Tgfb3*^+/+^ kidneys.

Our lipidomic analysis indicated increased levels of palmitic acid, glycerophospholipids, sphingolipids including ceramide (42:1), diacylglycerol and medium-chain triglycerides in *Tgfb3*^+/−^ kidneys, which are toxic intermediates that accumulate when β-oxidation is impaired and are also known to mediate insulin resistance and fibrosis. A complementary metabolomic enrichment overview showed that the main pathways deregulated upon *Tgfb3* downregulation were the tricarboxylic acid cycle and the mitochondrial electron transport chain. Indeed, all tested oxidative phosphorylation genes and some mitochondrial dynamics genes such as *Mfn1* and *Opa1* were downregulated in *Tgfb3*^+/−^ kidneys, with no changes in the total mitochondrial number, suggesting that the mitochondrial alterations could be functional. Furthermore, close inspection of *Tgfb3*^+/−^ kidneys showed marked morphological alterations including altered cristae structure and onion-like-shaped mitochondria, which have been reported to be pathological (Chaanine, 2019). These findings might be associated with the increase in ROS in *Tgfb3*^+/−^ mice and reinforce the idea of mitochondrial dysfunction. Further analysis of the enzymatic activity of critical enzymes involved in lipid metabolism are needed to explore the pathways linking the lack of *Tgfb3* to the metabolic deregulation and lipid accumulation.

Further *in vitro* experiments in different cell types and more studies in kidney-specific knockout animal models are necessary to better understand the underlying mechanisms involved in the phenotype associated with *Tgfb3* deficiency. The present results should be interpreted within the context of their limitations. However, and similar to other biological parameters such as oxidative stress and inflammation, we believe that the balance between the different isoforms of the TGFβ family is likely key for correct physiological functioning of fibrosis. As elegantly shown in dermal fibroblasts, fibrosis occurs when there is a lack of either TGFβ1 or TGFβ3; however, when the concentration of the two isoforms is similar, collagen levels are close to physiological levels (Shah et al., 1995). In the same study, the authors concluded that TGFβ3 may have an important role in controlling the fibrotic effects of TGFβ1 and in fine-tuning the synthesis of ECM.

**Fig. 7.**
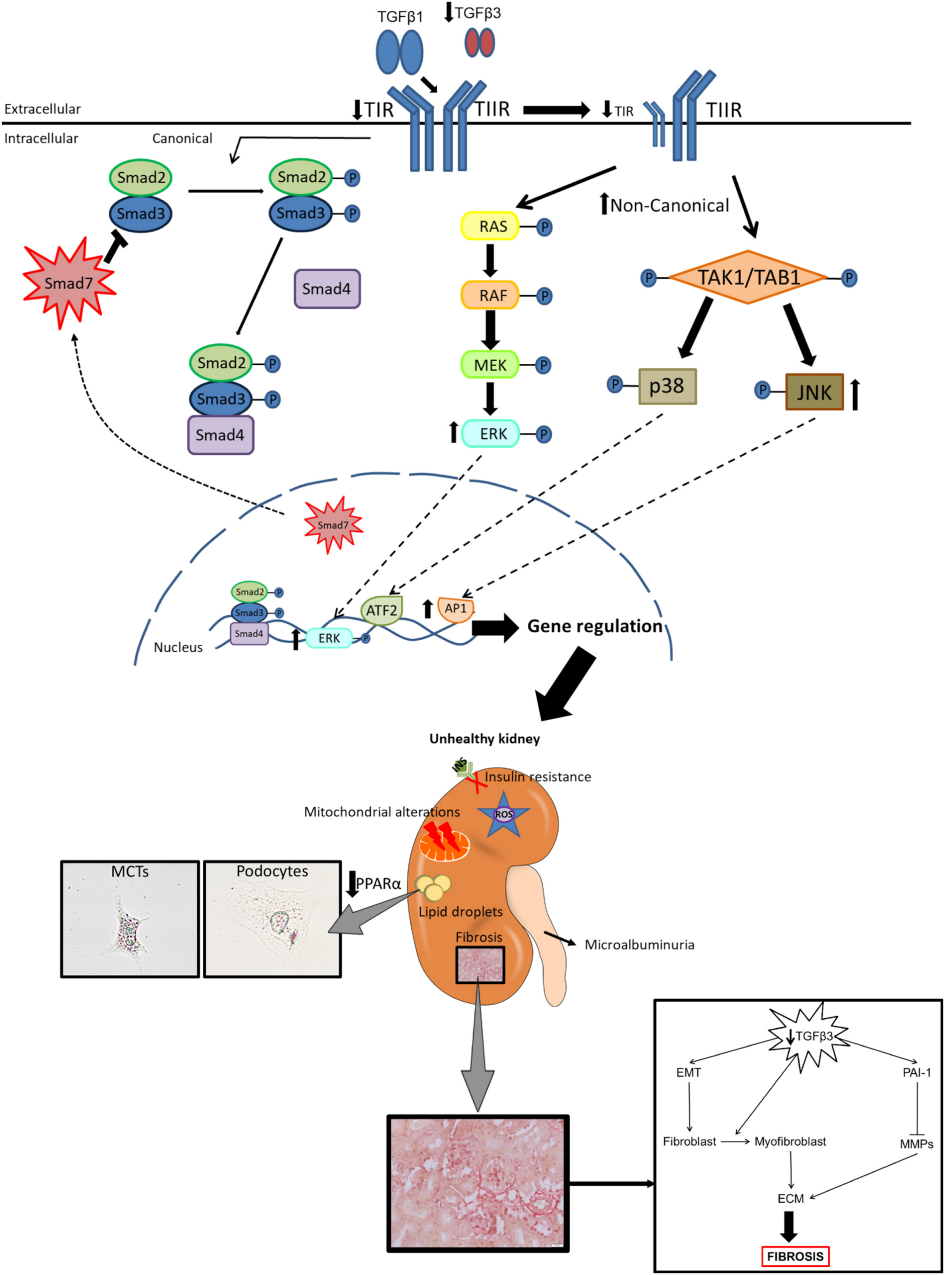
**TGFβ3 plays a key role in the physiopathology of the kidney.** Reduced TGFβ3 levels lead to a higher TGFβ3/TGFβ3 ratio and lower TIR levels. Because TGFβ1 and TGFβ3 share their receptors, lower levels of TGFβ3 mean higher bioavailability of the TIR and TIIR receptors to TGFβ1. Without TGFβ3 present to counteract the actions of TGFβ1, TGFβ1 overactivates their downstream pathways – SMAD2/3, ERK1/2 and JNK – leading to renal damage. The decrease in TIR levels leads to a preference in the activation of the non-canonical pathways over the canonical SMAD2/3 pathway. As a result, *Tgfb3*^+/−^ male mice show albuminuria, loss of renal function, lipid accumulation, insulin resistance, fibrosis, oxidative stress and mitochondrial alterations. Note that the podocyte image is reproduced from Fig. 3. ECM, extracellular matrix; EMT, epithelial–mesenchymal transition; MCT, mouse proximal tubular renal epithelial cells; MMP, metalloproteinase; TIR, TGFβ type I receptor; TIIR, TGFβ type II receptor.

We hypothesize that the mechanism responsible for the phenotype observed in our study could be the linked to the fact that TGFβ1 and TGFβ3 share the same receptors – TIIR, which recruits and activates TIR (Tzavlaki and Moustakas, 2020). Receptor activation requires the binding of ligand dimers, but these dimers may not necessarily need to be homodimeric. Accordingly, TIIR–TIR receptors can theoretically be activated upon binding of the homodimer TGFβ3, homodimer TGFβ1 or even by the heterodimer TGFβ1–TGFβ3 (Cheifetz et al., 1988; Huang et al., 2011; Tzavlaki and Moustakas, 2020). When TGFβ3 levels decrease, fewer TGFβ3 homodimers and, theoretically, fewer TGFβ1–TGFβ3 heterodimers would be available, and thus the TGFβ1 homodimers would be the prevalent ligands. In our model, the decrease in the abundance of TGFβ3 results in an imbalance in the TGFβ1/TGFβ3 ratio, and, although TGFβ1 is not increased as a compensatory mechanism, the bioavailability of TGFβ1 for the receptors could be significantly increased, possibly overactivating canonical SMAD2/3 signaling and non-canonical MAPK ERK1/2 and JNK downstream pathways (Gu et al., 2020). This hypothesis is supported by the evident increase, both *in vivo* and *in vitro*, in the phosphorylation of ERK and JNK pathways in our study. Activation of MAPK ERK1/2 and JNK pathways is known to be involved in the development of insulin resistance and in the regulation of mitochondrial activity (Guebre-Egziabher et al., 2013; Wortzel and Seger, 2011; Zeke et al., 2016; Zhao et al., 2019). In our model, we failed to observe an increase in the phosphorylation of SMAD2/3; however, *Smad7* mRNA levels were significantly decreased, suggesting that there is a lack of inhibition of this pathway. Thus, as suggested by others (Gu et al., 2020), the imbalance between SMAD2/3 and SMAD7 signaling would explain the EMT and the increased production of αSMA and collagens in our model (Fig. 7). A plausible explanation for why cells with diminished levels of TGFβ3 signal preferentially through the MAPK pathways rather than through the canonical SMAD2/3 pathway could be linked to the downregulation of TIR that we observed in *Tgfb3^+/−^* mice and in siTGFβ3 podocytes. It has been shown that cells tend to activate ERK1/2 when the bioavailability of TIIR is higher than that of TIR (Bandyopadhyay et al., 2011).

In conclusion, defects in TGFβ3 at the renal level result in (1) fibrosis associated with EMT, (2) defective maintenance of the glomerular filtration barrier with increased albuminuria and lower mGFR, (3) abnormal lipid metabolism associated with lipid accumulation, (4) insulin resistance and (5) mitochondrial dysfunction in male mice. Overall, our study provides evidence that renal TGFβ3 plays an important role in the maintenance of renal homeostasis, likely by opposing or compensating the role of TGFβ1 through competition for their common receptors. This could explain the lack of success in the use of non-isoform-specific TGFβ antibodies as therapies and may open new avenues in the field of fibrosis. As fibrotic processes seem to be similar and involve the TGFβ family in many organs, these results could help us to understand other fibrotic diseases.

## MATERIALS AND METHODS

### Animals

The generation of the original animal model has been published (Proetzel et al., 1995). Male and female C57/Bl6J mice heterozygous for a null mutation in *Tgfb3* (*Tgfb3*^+/−^) and wild-type littermates (*Tgfb3*^+/+^) derived from Martinez's laboratory at the Universidad Complutense in Madrid (Spain) were used for all experiments (Martínez-Sanz et al., 2008). Mice were housed in a temperature-controlled room with 12-h light-dark cycles and were placed on chow diet at 4 weeks of age (10% of fat; D12450B, Research Diets) with food and water available *ad libitum*. Mice were sacrificed at 1, 4 and 16 months of age, and their kidneys were rapidly dissected, weighed, and snap-frozen or processed with formalin for subsequent analysis. Serum was collected for biochemical measurements. All animal protocols used in this study were approved by the Research Ethics Committee of the Universidad Rey Juan Carlos and complied with relevant animal welfare laws, guidelines and policies.

For insulin sensitivity analysis, mice were fasted overnight (14 h) and then intraperitoneally injected with human insulin (Actrapid; Novo Nordisk) at 10 U/kg body weight or saline. Kidneys were harvested 5 min later and snap-frozen for protein extraction.

### Measurement of urine and serum parameters

Mice were placed in metabolic cages for 24 h. Urine was collected for measurement of urea, albumin and creatinine excretion, allowing the calculation of the ACR. Sera collected upon sacrifice were analyzed for triglycerides, total, low- and high-density lipoprotein (LDL and HDL, respectively) cholesterol, non-esterified fatty acids (NEFAs) and total protein. All parameters were measured at the Instituto de Investigación Sanitaria de Navarra, Pamplona, Spain. PAI-1 levels were measured with Bioplex kits (Bio-Rad). The glomerular filtration rate (GFR) was measured using the iohexol plasma clearance method (Luis-Lima et al., 2016).

### Blood pressure measurements

Blood pressure was assessed using a computerized non-invasive tail-cuff sphygmomanometer (NIPREM 645, Cibertec) on conscious 4-month-old mice on alternate days for 2 weeks. An average of ten measurements was registered per animal and day.

### Cell culture

We established a conditionally immortalized mouse podocyte cell line in collaboration with the Coward laboratory (Bristol University, Bristol, UK). Cells were maintained in RPMI medium supplemented with 10% fetal bovine serum and penicillin (100 U/ml) and streptomycin (100 μg/ml). The podocyte cell line proliferates at 33°C and becomes quiescent and differentiates when thermoshifted to 37°C. Differentiation requires 10–14 days.

MCTs were provided by Dr Ricardo J. Bosch (Universidad de Alcalá, Madrid, Spain) (Izquierdo et al., 2006). Cells were maintained as above at 37°C.

Knockdown of *Tgfb3* was achieved using siRNA-mediated silencing (siTGFβ3). Cells (podocytes and MCTs) were transfected with 40 nM of an siRNA specific for *Tgfb3* (sc-39805; Santa Cruz Biotechnology) or the corresponding scramble control (sc-37007; Santa Cruz Biotechnology) using Lipofectamine 2000 (Invitrogen). Cells were used for experiments 24 h after transfection.

### Quantification of active and total TGFβ

A quantitative bioassay for TGFβ based on its ability to induce PAI-1 expression was used for quantification of active and total TGFβ as described previously (Khan et al., 2012; Van Waarde et al., 1997). Mink lung epithelial cells (MLECs) were stably transfected with an expression construct containing a truncated PAI-1 promoter fused to the firefly luciferase reporter gene (MLEC-PAI1 cells). MLEC-PAI1 cells were generously provided by Dr Kampinga (University of Groningen, Groningen, The Netherlands) Addition of recombinant TGFβ (0.2 to >2 ng/ml) to the transfectants resulted in a dose-dependent increase in luciferase activity in the kidney lysates. Briefly, MLEC-PAI1 cells were cultured at 37°C in Dulbecco's modified Eagle medium (DMEM)-high glucose (4500 mg/l) supplemented with 10% fetal calf serum, L-glutamine (2 mM), penicillin (100 U/ml), streptomycin (100 mg/ml) and Geneticin (250 mg/ml).

MLEC-PAI1 cells (40,000 cells) were cultured in 100 µl culture medium per well in a 96-well tissue culture dish and were allowed to attach for ±3 h. In the meantime, in order to evaluate the amount of activated TGFβ, 30 μl kidney protein lysate (100–300 μg protein) was acid activated using 200 μl DMEM+0.1% pyrogen-poor bovine serum albumin and 10 μl of 1 M HCl. Kidney lysates were rocked for 1 h at 4°C. The acid activation was stopped by neutralization with 250 μl of DMEM+0.1% pyrogen-poor BSA and 10 μl of 0.75 M NaOH. When cells had attached, the medium was replaced with 100 µl activated or non-activated kidney lysates followed by incubation for 20 h at 37°C. Cells were then lysed with 100 µl lysis buffer (25 mM H_3_PO_4_–Tris buffer pH 7.8, 10 mM MgCl_2_, 1% Triton X-100, 15% glycerol and 1 mM EDTA) and frozen at −20°C for at least 2 h. Then, the luminescence in light units (RLU) of 150 µl cells lysate was measured from each well using a GloMax Multi Detection System E7041 (Promega). Luminescence was measured for 10 s following the addition of 100 µl substrates (1.25 mM ATP and 87 mg/ml luciferin). Results were interpolated in a standard curve built with known concentrations of recombinant TGFβ.

### RNA preparation and quantitative RT-PCR

Kidney RNA extraction, quantification and retrotranscription was performed as described (Martinez-Garcia et al., 2012). cDNA was also prepared from the conditionally immortalized mouse podocyte cell line, used for *in vitro* experiments. All quantitative RT-PCR assays were performed in duplicate for each sample for different genes. β-actin, *36B4*, *18S* and *B2M* were used as housekeeping genes. To validate housekeeping genes, we used the BestKeeper software tool (Pfaffl et al., 2004). All primers are listed in Tables S1 and S2.

### mtDNA copy number

mtDNA copy number was determined using the ratio between mtDNA and nDNA, as described (Quiros et al., 2017).

### Flow cytometry

Superoxide production was determined using a fluorometric assay with dihydroethidium (2HE) (37291; Sigma-Aldrich), as previously described (Martínez-García et al., 2015). Intracellular lipids were measured by a fluorometric assay using Bodipy (D3922; Thermo Fisher Scientific). The presence of podocin, insulin receptor and CD36 on the cell membrane was evaluated with specific antibodies (Table S3). Flow cytometry was performed on a Beckman Coulter Cytomics FC500 MPL cytometer. All measurements were done on whole kidney digested with collagenase (*Clostridium histolyticum*, Sigma-Aldrich).

### Western blotting

Protein extraction, quantification and western blotting were performed as described (Martinez-Garcia et al., 2012). Blots were visualized using a chemiluminiscent detection system (Clarity Western ECL, Bio-Rad) and scanned by ChemiDoc (Bio-Rad). Protein band density was measured using ImageJ 1.45 software (National Institutes of Health, Bethesda, MD, USA). All antibodies used are listed in Table S3.

### Immunohistochemistry

Renal paraffin sections (4 μm) were stained for α-SMA and nitrotyrosine. The protocol was carried out as described (Jha et al., 2014; Martinez-Garcia et al., 2012). All sections were examined under a light microscope (Zeiss ICS Standard 25) and digitized. For quantification of the proportional staining intensity, ten glomeruli were analyzed using ImageJ 1.45 software. All antibodies used are listed in Table S3.

### Histological staining and glomerular measurement

Renal paraffin sections (4 μm) were stained with Hematoxylin and Eosin (H&E) and Picrosirius Red. Frozen renal sections (7 μm) were used for Oil Red O staining. Glomerular images were digitized, and glomerular areas were calculated using Aperio ImageScope software (Leica Biosystems). To calculate the glomerulosclerosis index, glomerulosclerotic injury was graded based on the severity of glomerular damage, as previously reported (Lassila et al., 2004). Twenty glomeruli per kidney were assessed in a blinded fashion. Six kidneys were investigated per group.

### Immunofluorescence

Podocytes were mounted on cover slips and fixed with 4% paraformaldehyde. After blocking, cells were first incubated with antibodies against CD36 and insulin receptor (Table S3) and then with Alexa Fluor 488 (green) or Alexa Fluor 594 (red) secondary antibodies. Some samples were incubated without the primary antibody to serve as negative controls. The nuclei were visualized using 4′,6-diamidino-2-phenylindole (DAPI). Images were taken with an inverted fluorescence microscope (ECLIPSE 90i, Nikon Instruments Europe B.V.) or a confocal microscope LSM710 (Zeiss, Germany). Images of podocytes were scored by two independent blinded observers, who scored at least 100 cells per condition.

### Transmission electron microscopy

Kidney tissue was fixed in 2.5% glutaraldehyde, postfixed in 1% osmium tetroxide, dehydrated with an increasing concentration of ethanol and embedded in epoxy resin. Renal ultrathin sections were processed as described (Martinez-Garcia et al., 2012). Foot process width and glomerular basement membrane thickness were measured using ImageJ software, as previously described (Mallipattu et al., 2013). All mitochondrial parameters were measured using Fiji software (National Institutes of Health). Ten measurements per kidney and four kidneys were investigated per experimental group.

### Metabolomics and lipidomics study

#### Sample preparation

Pulverized kidneys were stored at −80°C until extraction. The method used for extraction of kidney samples was previously validated for tissue (Martinez-Garcia et al., 2012; Naz et al., 2013). Approximately 60 mg of pulverized kidney from each sample was used for the study. MeOH: H2O (1:1) was added to the samples as solvent in the proportion of 1:10 tissue/solvent, and the samples were then homogenized with a TissueLyser LT equipment (Qiagen, Germany). Hydrophobic compounds were extracted adding methyl-terc-butyl-ether (MTBE) to the homogenates (MeOH: MTBE 80:20). Then, 90 µl of the supernatant of each sample was transferred to chromatography vials for liquid chromatography–mass spectrometry (LC-MS) analysis and 300 µl of the supernatant of each sample was transferred to chromatography vials for gas chromatography–mass spectrometry (GC-MS) analysis. Derivatization was performed in samples for GC-MS analysis prior to analytic analysis. Samples were encapsulated individually and stored at −20°C until analysis.

Quality control (QC) samples were prepared independently and in parallel with the samples, combining the surplus of each supernatant sample after homogenization.

#### LC-MS and GC-MS analytical analyses

LC-MS analysis was performed in a UHPLC-QTOF-MS equipment with a HPLC 1290 Infinity II (Agilent Technologies) liquid chromatography system coupled to a 6545-ESI-QTOF/MS (Agilent Technologies) mass spectrometry equipment in positive and negative polarization. GC-MS analysis was performed in gas chromatography equipment from Agilent Technologies (7890B GC) mounted with a Gerstel Autosampler (MPS) coupled to an EI-QTOF-MS mass spectrometer (7250 MS, Agilent Technologies). Analyses were performed as previously described (Cala et al., 2018; Medina-Gomez et al., 2009).

QC samples were analyzed throughout the analytical measurements, providing a measurement of stability, performance and reproducibility of the system and the measurements obtained in the analyses.

#### Signal processing

LC-MS signals were processed with MassHunter Profinder (8.0.0). The Molecular Feature Extraction (MFE) tool was used to clean background noise and ions not related to identities and was configured to find co-eluting adducts with the same features. The Recursive Feature Extraction (RFE) tool was used to improve the quality of the final compound list using spectra built for each feature with MFE, re-extracting file batches obtained with MFE. Specific conditions and software configurations have been previously defined (Gonzalez-Riano et al., 2017).

GC-MS signals were processed with MassHunter Unknown Analysis (7.0.0). Chemical allocation of compounds was performed by comparing the extracted spectrum from deconvolution and retention times obtained for each compound using the Fiehn library (2018 version), an in-house library created by CEMBIO (Kind et al., 2009) and NIST (2.2). Data were aligned with MassHunter Professional (B.12.1), and MassHunter Qualitative (B.08.0.0) was used to assign target ions and perform signal integration. Specific conditions and software configurations have been previously defined (Gonzalez-Riano et al., 2017).

#### Data treatment

In LC-MS and GC-MS analyses, missing values detected in the final list of features obtained after signal processing were treated applying the k-nearest neighbors’ (KNN) algorithm. GC-MS raw data were normalized using the joint and individual variation explained (JIVE) algorithm (Kuligowski et al., 2015). LC-MS raw data did not need to be normalized because the 1:10 tissue/solvent ratio was maintained in the initial homogenization of the kidney samples. Therefore, the same theoretical amounts of lipids were extracted per mg of tissue in each sample. Features were filtered with relative s.d. (RSD) calculated for QC samples in LC-MS and GC-MS analyses. Features that presented an RSD of 30% were selected.

#### Statistical analysis

Univariate (UVA) and multivariate (MVA) analyses were performed for LC-MS and GC-MS analyses, as previously described (Wang et al., 2011). In univariate analysis, a non-parametric method through Mann–Whitney U test was applied. *P*-values were obtained for each feature in UVA. MVA was performed with SIMCA-P 15.0 (Umetrics). An orthogonal partial least squares discriminant analysis (OPLS-DA) model was built between *Tgfb3*^+/+^ and *Tgfb3*^+/−^, then the variable influence of projection (VIP) used to select the significant features obtained in the comparison between groups was obtained for each feature. Metabolites with *P*≤0.05 and/or VIP>1 were selected as significant.

#### Annotation of significant features selected in LC-MS analysis

The significant metabolites obtained in LC-MS analysis were annotated by tandem mass spectrometry (MS/MS) using published spectral libraries and resources (Godzien et al., 2015).

Enrichment analysis and heatmaps were created using Metaboanalyst 4.0 (Chong et al., 2018). LC-MS and GC-MS data are available at the Metabolomics Workbench platform (access number ST001303; https://www.metabolomicsworkbench.org/data/DRCCMetadata.php?Mode=Study&StudyID=ST001303).

### Statistical analysis

All parameters were analyzed using GraphPad Prism 7 software (GraphPad Software, La Jolla, CA, USA). One-way ANOVA followed by post hoc testing (Bonferroni correction) were conducted for multiple comparisons of the means. For comparisons between two groups, we used unpaired Student's *t*-test with Welch's correction; *P*<0.05 was considered statistically significant. Results are expressed as the mean±s.e.m. For the mGFR, data are represented as median±interquartile range (IQR) and the Mann–Whitney U test was performed.

## Supplementary Material

Supplementary information
