# Supplementary material for: Transforming growth factor β3 deficiency promotes defective lipid metabolism and fibrosis in murine kidney
Source: Dis Model Mech. 2021 Oct 1;14(9):dmm048249. doi: 10.1242/dmm.048249 (PMC8489029; doi:10.1242/dmm.048249)
Supplement: Supplementary information [file dmm-14-048249-s1.pdf]

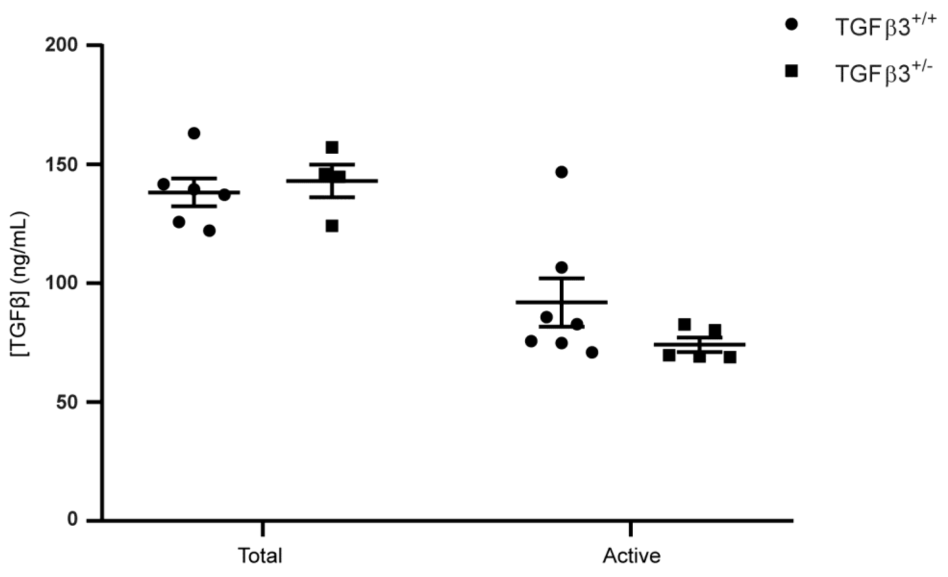

Fig. S1. Concentration of total and active TGFβ in kidney extracts of *Tgfβ3*$^{+/-}$ and *Tgfβ3*$^{+/+}$ mice. Quantitative bioassay for active and total TGFβ based on its ability to induce PAI-1 expression using mink lung epithelial cells (MLEC) stably transfected with a truncated PAI-1 promoter fused to the firefly luciferase reporter gene. Addition of recombinant TGFβ (0.2 to > 2 ng/mL) to the transfectants resulted in a dose-dependent increase in luciferase activity in the whole 4-month-old male kidney lysates (n=5–7). Data are shown as mean ± SEM. A t-test was performed with Welch's correction. * $p < 0.05$ versus the control.

Disease Models & Mechanisms • Supplementary information

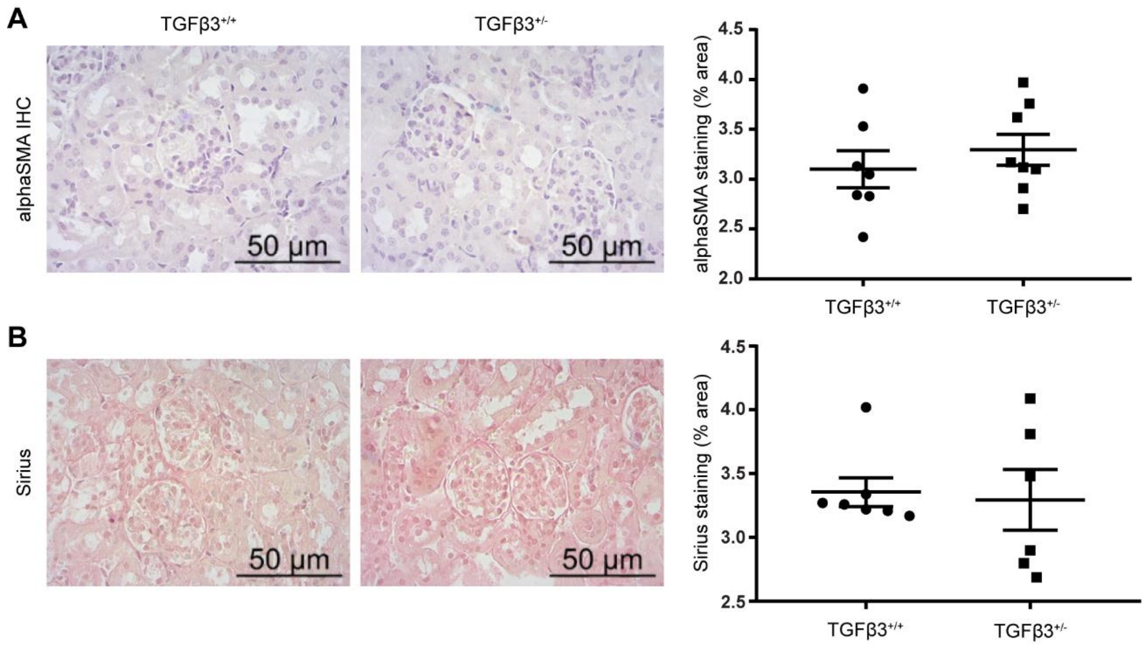

Fig. S2. Examination of renal fibrosis in female *Tgfβ3*$^{+/-}$ and *Tgfβ3*$^{+/+}$ mice. (A) α-SMA immunohistochemistry and (B) Picrosirius staining in kidney from 4-month-old female mice (n=6–8). Images were taken at a magnification of 400×. α-SMA, α smooth muscle actin; IHC, immunohistochemistry. Data are shown as mean ± SEM. A t-test was performed with Welch's correction. ***** $p < 0.05$ versus the control.

Disease Models & Mechanisms • Supplementary information

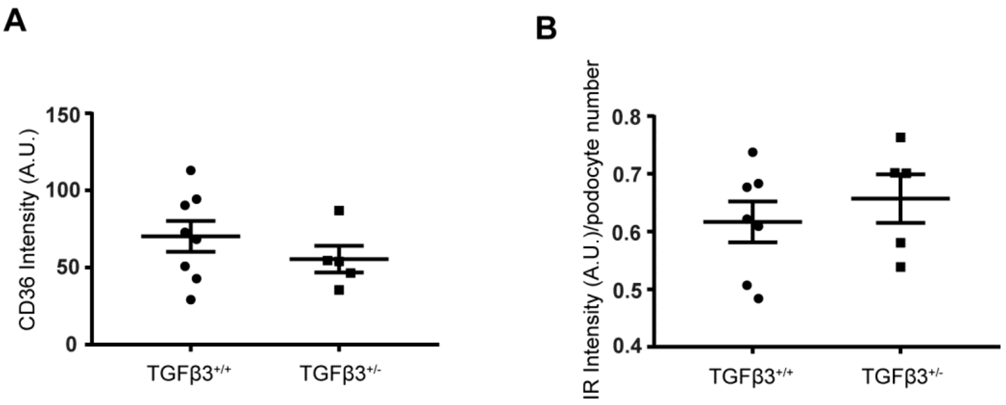

Fig. S3. Lipid metabolism in *Tgfβ3*$^{+/-}$ and *Tgfβ3*$^{+/+}$ kidney. (A) CD36 staining intensity measured by flow cytometry (n=5–8) in whole kidney of 4-month-old male mice and (B) Insulin receptor (IR) staining measured by flow cytometry in kidney from 4-month-old male mice (n=5–7). Data are shown as mean ± SEM. A t-test was performed with Welch's correction. * $p < 0.05$ versus the control.

Disease Models & Mechanisms • Supplementary information

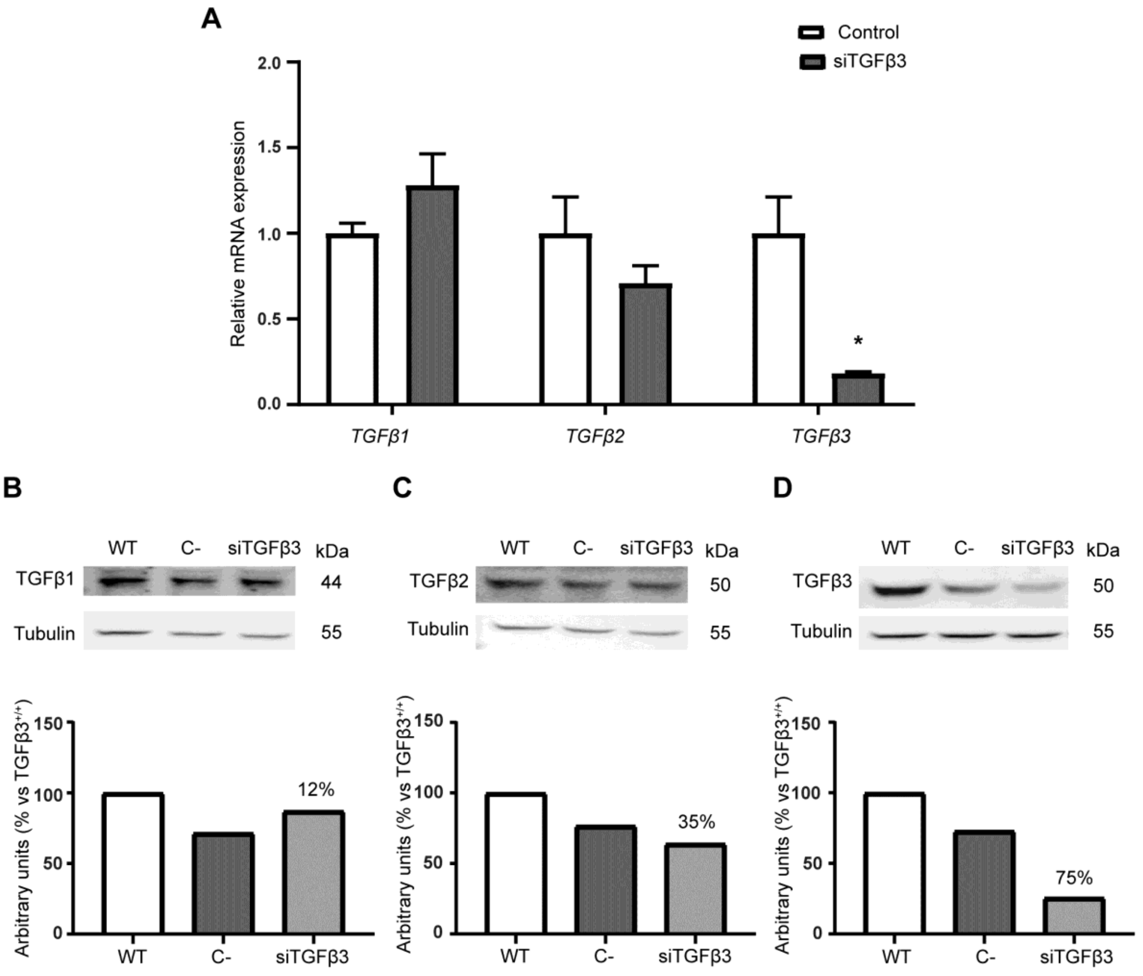

Fig. S4. TGFβ expression in siTGFβ3 podocytes. (A) Relative mRNA expression of *Tgfβ1*, *Tgfβ2* and *Tgfβ3* in control and siTGFβ3 podocytes (n=3). (B–D) Western blot quantification of TGFβ1, TGFβ2 and TGFβ3 in control and siTGFβ3 podocytes. Data are shown as mean ± SEM. A t-test was performed with Welch's correction. * $p < 0.05$ versus the control.

Disease Models & Mechanisms • Supplementary information

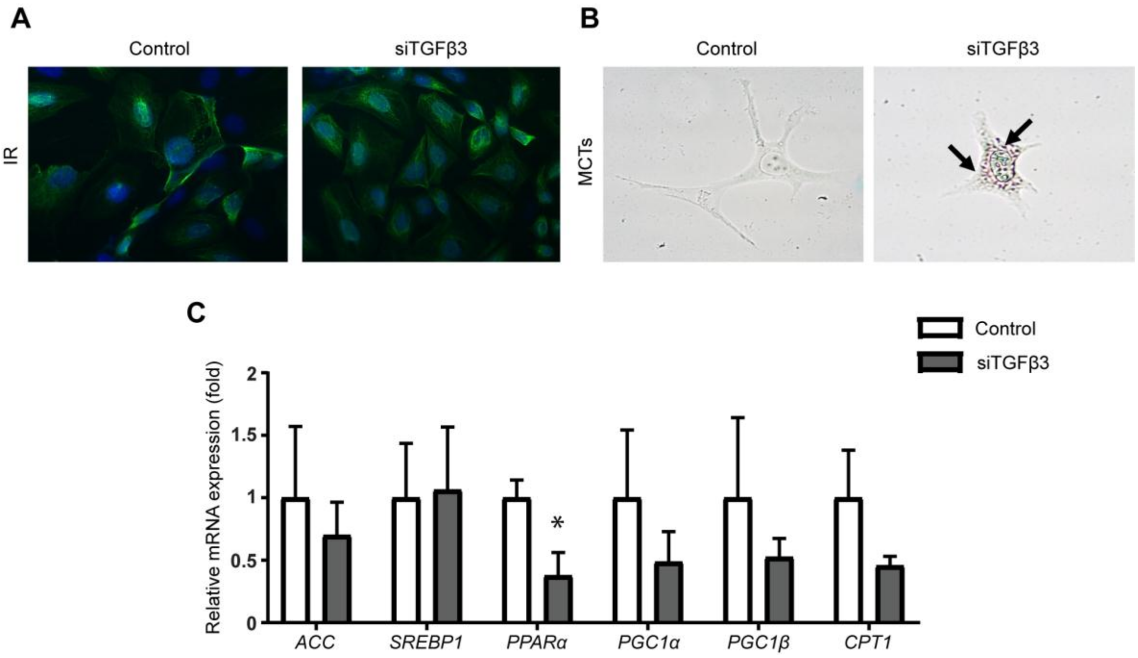

Fig. S5. *In vitro* experiments in siTGFβ3 podocytes and siTGFβ3 MCTs. (A) Immunofluorescence for IR in control and siTGFβ3 podocytes (n=3). (B) Oil Red staining (400× magnification) in control and siTGFβ3 mouse proximal tubular epithelial cells (MCTs) (n=3). (C) Relative mRNA expression of lipid metabolism genes in control and siTGFβ3 MCTs (n=3). Images were taken at a magnification of 400×. Black arrows mark red dots representing lipid droplets. Data are shown as mean ± SEM. A t-test was performed with Welch's correction. ***** $p < 0.05$ versus the control.

Disease Models & Mechanisms • Supplementary information

Table S1. SYBR Primers

Gene	Forward	Reverse
36B4	AGATGCAGCAGATCCGCAT	GTTCTTGCCCATCAGCACC
ACC	TGACAGACTGATCGCAGAGAAAG	TGGAGAGCCCCACACACA
B2M	ACTGATACATACGCCTGCAGAGTT	TCACATGTCTCGATCCCAGTAGA
CPT1	CACGAAGAGCTAAACTTG	TATGAGAGGGTGCTGCTT
E-cadherin	CACCTGGAGAGAGGCCATGT	TGGGAAACATGAGCAGCTCT
Mfn1	AATTAACTTCCTGCGTTGCTTT	GATGGAACCCACCAAACCCA
mt12S	TTGGTAAATTTCGTGGCAGCCACC	CAGTTTGGGTCTTAGCTGTCGTGT
mtAtp6	CAGTCCCCTCCCTAGGACTT	TCAGAGCATTGGCCATAGAA
mtCo1	CTCGCCTAATTTATTCCACTTCA	GGGGCTAGGGGTAGGGTTAT
mtCo2	ACCTGGTGAACTACGACTGCTAGA	TGCTTGATTTAGTCGGCCTGGGAT
mtCytB	ACCAATCTCCCAAACCATCA	TCCAGAGACTTGGGGATCTAAC
mtND1	GGGATAACAGCGCAATCCTA	ATCGTTGAACAAACGAACCA
N-cadherin	ATGTGCCGGATAGCGGGAGC	TACACCGTGCCGTCCTCGTC
OPA1	TGCCCTAACCATTTCAGAGGG	TCGAGAGCTCCCATCCCTAC
PGC1α	AACCACACCCACAGGATCA	CTCTTCGCTTTATTGCTCCATGA
PGC1β	CTGACGTGGACGAGCTTTCA	CCTTCAGAGCGTCAGAGCTT
PPARα	CCTCAGGGTACCACTAGGGAGT	GCCCGAATAGTTCGCCGAAA
SREBP1	GCCATGGATTGCACATTTGA	GGCCCGGGAAGTCACTG
SMAD7	AAGTGTTCAGGTGGCCGGATCTCAG	ACAGCATCTGGACAGCCTGCAGTTG
TFAM	CAGGAGGCAAAGGATGATTC	CCAAGACTTCATTTCATTGTCG
TGFβ1	GGATACCAACTATTGCTTCAG	TGTCCAGGCTCCAAATATAG
TGFβ2	GAGATTTGCAGGTATTGATGG	CAACAACATTAGCAGGAGATG
TGFβ3	GGTTACTATGCCAACTTCTG	CACATAGTACAAGATGGTCAG
β-catenin	ACTGCTGGGACTCTG	TGATGGCGTAGAACAG
TIR	CCTGAAGTTCTAGATGATT	CTTCATGGATTCCACCAAT
TIIR	CCAGGATGAATGTGGAAAAC	TAATCCTTCACTTCTCCCAC
**gDNA**		
g16S	CCGCAAGGGAAAGATGAAAGAC	TCGTTTGGTTTCGGGGTTTC
gHK2	GCCAGCCTCTCCTGATTTTAGTGT	GGGAACACAAAAGACCTCTTCTGG
gND1	CTAGCAGAAACAAACCGGGC	CCGGCTGCGTATTCTACGTT

Table S2. TaqMan Primers

Gene	Forward	Reverse	Probe
18S	CGGCTACCACATCCAAGGAA	GTCGGAATTACCGCGGCT	GAG GGC AAG TCT GGT GCC AG
F4/80	CAGATACAGCAATGCCAAGCA	GATTGTGAAGGTAGCATTCACAAGTG	TGCAGGGCAGGGATCTTGGTTATGC
β-actin	GCTCTGGCTCCTAGCACCAT	GCCACCGATCCACACAGAGT	ATCAAGATCATTGCTCCTCCTGAGCGC

Disease Models & Mechanisms • Supplementary information

Table S3. Antibodies

Gene	Manufacturer	Reference	Dilution
TGFβ1	Abcam	ab92486	1:400
TGFβ2	Abcam	ab36495	1:1000
TGFβ3	Abcam	ab15537	1:200
Phospho-JNK	Cell Signaling	9251	1:500
Total JNK	Cell Signaling	9258	1:1000
Phospho-ERK1/2	Cell Signaling	9106	1:1000
Total ERK 1/2	Cell Signaling	4695	1:1000
Phospho-SMAD2/3	Cell Signaling	8828	1:500
Total SMAD2/3	Cell Signaling	3102	1:1000
Phospho-AKT	Cell Signaling	4051	1:1000
Total AKT	Santa Cruz	sc8312	1:1000
Tubulin	Sigma-Aldrich	T5168	1;5000
Podocin	Abcam	ab65291	1;60
Nitrotyrosine	Millipore	06-284	1:100
α-SMA	Dako	M0851	1:100
CD36	Santa Cruz	sc7309	1:50
IRα	Biorbyt	650068	1:50

Table S4. General and metabolic parameters of female *Tgfβ3*$^{+/+}$ and *Tgfβ3*$^{+/-}$ mice at 1 and 4 months of age

		1 month		4 months	
	**Units**	***Tgfβ3*$^{+/+}$**	***Tgfβ3*$^{+/-}$**	***Tgfβ3*$^{+/+}$**	***Tgfβ3*$^{+/-}$**
**Total body weight**	g	12.63±0.32	12.43±0.33	20.87±0.60	20.78±0.49
**Glucose**	mg/dL	10.38±0.54	11±0.28	10.9±1.21	10.35±0.96
**Triglycerides**	md/dL	161.2±9.67	150.34±22.70	198.14±11.25	176.08±16.93
**Total Cholesterol**	mg/dL	108.34±3.25	93.03±10.47	102.77±6.68	109.48±13.06
**LDL**	mmol/L	0.71±0.05	0.66±0.05	0.78±0.21	0.52±0.07
**HDL**	mmol/L	1.80±0.06	1.48±0.19	1.31±0.34	0.93±0.49
**Total Proteins**	g/dL	6.23±0.14	5.95±0.33	6.53±0.19	6.88±0.39
**NEFAs**	mmol/L	3.32±0.19	3.08±0.21	4.47±0.37	3.15±0.77
**Kidney weight**	g	0.06±0.003	0.06±0.009	0.097±0.004	0.11±0.003
**Glomeruli size**	μm^2	21550.38±2417.72	24545.9±5643.95	32671.98±7194.8	34103.97±3872.01
**Serum Albumin**	g/L	4.32±0.24	4.23±0.25	4.24±0.18	4.54±0.22
**ACR**	mg/mmol	5.05±1.09	5.93±0.71	24.75±5.23	17.003±1.08

Notes: LDL, low-density lipoprotein; HDL, high-density lipoprotein; NEFAs, non-esterified fatty acids; ACR; albumin-creatinine ratio. n=7–12 animals per group.

Disease Models & Mechanisms • Supplementary information
